# Supplementary material for: Association of OPRM1 and OPRD1 Polymorphisms with Pain and Opioid Adverse Reactions in Colorectal Cancer
Source: Pharmaceuticals (Basel). 2025 Feb 6;18(2):220. doi: 10.3390/ph18020220 (PMC11860135; doi:10.3390/ph18020220)
Supplement: Supplementary file 1 [file pharmaceuticals-18-00220-s001.zip › pharmaceuticals-3396640-supplementary.pdf]

Supplementary material

Table S1. Logistic regression model for ADR.

Variable	Coefficient (β)	Standard Error	z Value	95% Confidence Interval (CI)	p-value
Intercept<br><br>OPRM1 (rs1799971) = A/A	2.3514	0.5233	4.494	(1.33, 3.37)	7e-06 ***
OPRM1 (rs1799971) A/G	-0.4543	0.8106	-0.560	(-1.83, 0.92)	0.575
Intercept<br><br>OPRM1 (rs510769) = T/T	1.2528	0.8018	1.562	(0.21, 2.30)	0.118
OPRM1 (rs510769) C/C	1.4553	1.0845	1.342	(0.32, 2.95)	0.180
OPRM1 (rs510769) C/T	0.8675	1.0081	0.861	(-0.15, 2.05)	0.389
Intercept<br><br>OPRD1 (rs2236861) = A/A	17.5700	1978.0900	0.009	(-19.18, 54.32)	0.993
OPRD1 (rs2236861) A/G	-15.1200	1978.0900	-0.008	(-52.77, 22.53)	0.994
OPRD1 (rs2236861) G/G	-15.6200	1978.0900	-0.008	(-53.27, 22.03)	0.994

***p value < 0.05 is considered significant**

Table S2. Logistic regression model for pain.

Variable	Coefficient (β)	Standard Error	z Value	95% Confidence Interval (CI)	p-value*
Intercept OPRM1 (rs1799971) = A/G	1.8971	0.6191	3.064	(0.68, 3.12)	0.002
OPRM1 (rs1799971) A/A	0.4543	0.8106	0.560	(-1.14, 2.05)	0.575
Intercept OPRM1 (rs510769) = C/C	2.7081	0.7303	3.708	(1.27, 4.14)	0.0002
OPRM1 (rs510769) T/T	-1.4553	1.0845	-1.342	(-3.58, 0.67)	0.180
OPRM1 (rs510769) C/T	-0.5878	0.9522	-0.617	(-2.45, 1.28)	0.537
Intercept OPRD1 (rs2236861) = G/G	1.9459	0.4781	4.070	(1.01, 2.89)	4.7e-05
OPRD1 (rs2236861) A/A	15.6202	1978.0900	0.008	(-34.66, 65.91)	0.994
OPRD1 (rs2236861) A/G	0.4964	0.8787	0.565	(-1.23, 2.22)	0.572

***p value < 0.05 is considered significant**

Table S3. Logistic regression model for ADR analysis, incorporating the available MME data.

Variable	Coefficient	95% CI	p-value
Intercept	1.47e+7	(NA, NA)	0.995
OPRD1 (rs2236861) A/G	1.16e-7	(NA, 4.32e204)	0.995
OPRD1 (rs2236861) G/G	1.16e-7	(NA, 5.51e204)	0.995
MME	1.00e+0	(9.55e-1, 1.06e0)	0.875
Intercept	3.17	(0.354, 42.3)	0.326
OPRM1 (rs1799971) A/G	0.267	(0.0345, 1.74)	0.176
MME	1.00	(0.952, 1.07)	0.884
Intercept	1.43	(0.112, 21.6)	0.779
OPRM1 (rs510769) C/C	1.29	(0.0813, 20.6)	0.852
OPRM1 (rs510769) C/T	2.00	(0.168, 25.2)	0.571
MME	1.00	(0.940, 1.07)	0.960

MME: morphine milligram equivalents.
